# Trunnionosis in total hip arthroplasty: a review

**DOI:** 10.1007/s10195-016-0391-1

**Published:** 2016-02-11

**Authors:** Jaydev B. Mistry, Morad Chughtai, Randa K. Elmallah, Aloise Diedrich, Sidney Le, Melbin Thomas, Michael A. Mont

**Affiliations:** Rubin Institute for Advanced Orthopedics, Center for Joint Preservation and Replacement, Sinai Hospital of Baltimore, 2401 West Belvedere Avenue, Baltimore, MD 21215 USA

**Keywords:** Trunnionosis, Trunnion, Total hip arthroplasty, Corrosion

## Abstract

Trunnionosis is defined as wear of the femoral head–neck interface and has recently been acknowledged as a growing cause of total hip arthroplasty failure. Some studies have reported that it accounts for up to 3 % of all revisions. The exact cause of trunnionosis is currently unknown; however, postulated etiologies include modular junction wear, corrosion damage, and metal ion release. Additionally, implant design and trunnion geometries may contribute to the progression of component failure. In order to aid in our understanding of this phenomenon, our aim was to present the current literature on (1) the effect of femoral head size on trunnionosis, (2) the effect of trunnion design on trunnionosis, (3) localized biological reactions associated with trunnionosis, and (4) gross trunnion failures. It is hoped that this will encourage further research and interest aimed at minimizing this complication.

## Introduction

Trunnionosis is defined as the wear of the femoral head–neck interface and has been acknowledged as a source of total hip arthroplasty (THA) failure [1]. This phenomenon appears to have gained prevalence with newer THA implant designs, particularly when modularity was introduced. Modularity allows for a better intraoperative restoration of leg length and control of hip offset [2], but while this enables a more customized fit for the patient, it may have untoward effects. The modularity at times may play a role in increased wear and mechanical insufficiency at the trunnion, ultimately leading to revision [3]. In fact, trunnionosis is estimated to account for up to 3 % of all THA revision procedures [4, 5].

The exact cause of trunnionosis, which is likely multi-factorial, currently remains poorly understood. It is postulated that contributing factors include wear between metal-on-metal modular junctions [6], corrosion and fretting damage [7], and the release of metal ions or particulate debris from affected components [8]. Additionally, different implant designs and geometries have demonstrated a predisposition to trunnion failure [2].

Multiple studies have postulated on the causes of trunnionosis. Therefore, we will examine the current literature and collate the evidence on trunnionosis following THA. Specifically, we present the literature on (1) the effect of femoral head size on trunnionosis, (2) the effect of trunnion design on trunnionosis, (3) localized biological reactions associated with trunnionosis, and (4) gross trunnion failures.

## Methods

We performed a literature search using PubMed, EBSCO Host, and SCOPUS. We searched studies from inception of the respective databases up to September 2015 using various combinations of the following search terms—‘trunnion’, ‘THA’, ‘total hip arthroplasty’, ‘taper’, ‘wear’, ‘trunnionosis’, ‘hip’, ‘corrosion’, ‘fretting’, ‘ARMD’, ‘junction’, ‘ALTR’, ‘debris’, and ‘component’.

We reviewed 102 abstracts to determine articles that were appropriate for this review. We identified 70 potentially relevant articles for further evaluation. We excluded 45 articles that were not in English or not relevant to the topic. We then assessed the references of articles and found an additional 11 reports. We included four articles discussing femoral head sizes, four articles discussing trunnion design, six articles discussing local biological reactions, and four articles discussing gross trunnion failure.

## Discussion

### Large-diameter femoral heads

The introduction of large-diameter femoral heads was postulated to decrease component instability while also increasing the impingement-free range-of-motion. However, larger femoral heads have been considered to cause an increase in the effective horizontal lever arm, which imparts greater torsional forces at the head–neck junction [9] (Table 1).

**Table 1:** Summary of studies reporting femoral head size

References	Number of hip implants	Implant description	Results
Lavernia et al. [3]	Five models (via finite elemental analysis)	28-, 32-, and 40-mm heads	Trunnion stress increased with head size: 10.1, 12.1, 16.6 MPa
Bolland et al. [10]	199	38–58-mm heads	17 hips revised, 14 hips awaiting revision (all heads >40 mm)
Dyrkacz et al. [11]	74	28-mm head (59 hips)	Larger diameter group––greater correlation with head–neck corrosion (0.975 vs 0.502) and head–neck fretting (1.0 vs 0.366)
		36-mm head (15 hips)	
Matthies et al. [12]	110	>36-mm heads	Median volume of material loss at trunnion (0.29 mm^3^) was significantly less compared to female taper, femoral head, and acetabular bearing surface (*P* < 0.001). Correlation observed between corrosion score and volume of material loss (*ρ* = 0.72, (95 % CI 0.44–0.87, *P* < 0.001)

In a finite elemental analysis model assessing five trunnion–head junctions, Lavernia et al. [3] determined that not only was the area of maximum stress located on the medial aspect of the femoral neck, but also that the maximum stress in this area increased with larger head diameters. Maximum principal stresses for 28-, 32-, and 40-mm cobalt-chromium (CoCr) heads were 20.3, 36.0, and 43.8 megapascals (MPa), respectively. Stress increases within the trunnion were also computed. The trunnions for the 28-, 32-, and 40-mm CoCr heads exhibited stresses of 10.1, 12.1, and 16.6 MPa, respectively, in the central area of the trunnion adjacent to the femoral head. The authors concluded that stress levels were directly correlated to femoral head diameter. This may imply that the increased forces at the trunnion exerted by larger diameter heads play a role in component failure. Bolland et al. [10] published high failure rates after examining a cohort of 199 hips (185 patients) with large-diameter (>38 mm) hybrid THAs. At a mean follow-up of 62 months, 17 hips (8.5 %) had undergone a revision, with 14 more awaiting surgery. Between all hips that were revised or awaiting revision, the mean femoral head diameter was 46 mm (range 40–54 mm). During revision surgery, retrieved components displayed corrosion of the stem surface as well as increased wear at the head–neck junction. Dyrkacz et al. [11] found a significant difference in corrosion scores in the bore taper of a 36-mm head (15 hips) compared to a 28-mm head (59 hips) (*P* = 0.022) in a retrieval analysis to determine the effect of head size on corrosion and fretting in modular THA prostheses. When comparing the relationship between the heads and necks for corrosion damage, the 36-mm group demonstrated a greater correlation compared to the 28-mm group (0.975 vs 0.502). The 36-mm group also exhibited a greater correlation for head and neck fretting damage (1.0 vs 0.366).

Conversely, some studies have described how large-diameter femoral heads may not have a significant effect on damage at the trunnion–taper junction. In a retrospective study examining 110 large-diameter (>36 mm) THAs, Matthies et al. [12] demonstrated that the volume of material loss was negligible (<1 mm^3^) in the trunnions of all 36 femoral stems that were received. In fact, the median volume loss (0.29 mm^3^) was significantly less than that of the female taper, the femoral head, and acetabular-bearing surfaces (*P* < 0.001 for all). However, for the trunnions in this study, it should be noted that a significant positive correlation was observed between corrosion score and volume of material loss (*ρ* = 0.72, 95 % CI = 0.44–0.87, *P* < 0.001). Triantafyllopoulos et al. [13] conducted a retrieval analysis of 154 THAs. Ultimately, they found no association between femoral head size and the degree of fretting and corrosion on tapers (*P* = 0.247; *P* = 0.837, respectively) or trunnions (*P* = 0.471; *P* = 0.868, respectively). While this type of damage may be a regular occurrence in THAs, neither fretting nor corrosion was associated with femoral head size.

In summary, the association between large-diameter femoral heads and increased trunnion wear is inconclusive. While large femoral head may potentially cause elevated trunnion stresses, future studies should investigate if other factors, such as alloy composition, have greater responsibility.

### Trunnion design

The geometries of different trunnion designs may contribute to trunnionosis, as they influence torsional forces at the trunnion–taper interface [14]. Recently, trunnions have become shorter in length in an effort to increase the impingement-free range-of-motion (Table 2).

**Table 2:** Summary of studies reporting trunnion design

References	Number of hip implants	Implant description	Results
Tan et al. [15]	44	28-mm heads; six taper designs	Significantly greater corrosion scores within the trunnion base zone (*P* = 0.018)
Brock et al. [16]	104 female tapers, 11 stem trunnions	Short 12/14 trunnions versus long 11/13 trunnions	Higher rate of material loss with shorter trunnion compared to longer trunnion (0.402 vs 0.123 mm^3^/year; *P* = 0.035)
Porter et al. [4]	85	Stems released between 1983 and 2012; 10 different taper designs; five metal alloys from 16 manufacturers	Negative correlation between flexural rigidity (−0.23; *P* = 0.04) and trunnion length (−0.53; *P* < 0.001) with release date of the stem. Multiple regression analysis showed flexural rigidity (*β* = −0.17) and trunnion length (*β* = −0.51) were independently correlated with time
Nassif et al. [18]	40	Taper diameters: type one (eight hips), 11/13 (six hips), 12/14 (26 hips)	Higher fretting scores in 11/13 compared to type one tapers (*P* = 0.005)

Unfortunately, a shorter trunnion requires that its base sits within the taper of the femoral head, which may increase the likelihood of edge loading. Tan et al. [15] previously described this effect on the base in a retrieval analysis of 44 implants. When evaluating different regions of the trunnion, the authors noted significantly greater corrosion scores within the base zone (*P* = 0.018). They concluded that the base zone is subject to higher mechanical loading and greater torque forces compared to other regions. Brock et al. [16] retrieved 104 female tapers and 11 stem trunnions from 98 patients who experienced adverse reaction to metal debris. The authors used a co-ordinate measuring machine to assess for linear and volumetric wear of either shorter 12-mm/14-mm threaded trunnions or longer, smoother 11-mm/13-mm trunnions. They were able to identify significantly higher rates of material loss with the shorter, threaded 12-mm/14-mm trunnions compared to the longer, smooth 11-mm/13-mm trunnions (0.402 vs 0.123 mm^3^/year; *P* = 0.035). Porter et al. [4] conducted a retrieval analysis of 85 modular femoral stems released between 1983 and 2012 to determine how trunnion flexural rigidity and length have changed over time. They found a negative correlation between flexural rigidity (−0.23; *P* = 0.04) and trunnion length (−0.53; *P* < 0.001) with release date of the stem. Additionally, multiple regression analysis showed that flexural rigidity (*β* = −0.17) and trunnion length (*β* = −0.051) were independently correlated with time. Investigation of components in this study demonstrated that as new femoral stems were introduced, trunnions became less rigid and shorter.

Trunnion diameters have also been trending towards slimmer measurements in an effort to avoid impingement, i.e., from the older 14-mm/16-mm diameter to the more widely used 12-mm/14-mm diameter. It is thought that a decreased trunnion diameter translates to a reduced surface area for contact length and fitting, and therefore, increases the possibility for these complications [9, 17]. Contrary to this, a retrospective retrieval study by Nassif et al. [18] examined a small group of failed implants and revealed that thicker tapers with longer contact lengths were associated with greater fretting scores. Trunnions with 11-mm/13-mm tapers demonstrated significantly higher fretting scores compared to the narrower type one tapers (*P* = 0.005). This study also included 12-mm/14-mm tapers, but showed no significant association with fretting or corrosion scores. This particular investigation is limited because it examined a small group of failed implants from a heterogeneous cohort with respect to taper–trunnion geometry, alloy composition, implant manufacturer, and reason for revision.

In summary, many studies show how large femoral head diameters and shorter trunnion lengths might affect component damage. More research is necessary, as results concerning the effects of trunnion design still have to be considered inconclusive at present.

### Local biological reactions

Local soft-tissue reactions have been observed as a result of corrosion debris produced at the trunnion [19, 20]. The clinical and histological appearance seen in periprosthetic tissue reactions surrounding corroded trunnions is similar to that of adverse local tissue reactions (ALTRs) seen in defective metal-on-metal (MoM) [21, 22] and non-MoM [22, 23] bearings. Adverse biologic reactions related to metal components include necrosis, lymphocytosis, vasculitis, and production of exudates, pseudotumors, or sinuses [24–26]. Pseudotumors, which are non-malignant soft-tissue growths due to particulate debris irritation [27], have a histological appearance reflecting that of metal wear reactions and metal hypersensitivity. Many reports describing ALTRs note the use of implants containing cobalt, a metal that is known to have a more deleterious effect on macrophages compared to other metals found in implants [28]. These distinct tissue reactions have also been termed adverse reaction to metal debris (ARMD) [29] or aseptic lymphocyte-dominated vasculitis-associated lesions (ALVAL) [21]. It is possible that corrosion and ion release at the trunnion may be a provoking factor.

Several studies have described ALTRs related to trunnion wear (Table 3). In a case series examining ten THAs, Cooper et al. [30] described how the femoral head–neck junction in all patients contained a black, flaky material at the trunnion. Moreover, the surgeons encountered large amounts of white to brownish fluid, hypertrophic synovial tissue, and pseudotumors in several cases. One patient who demonstrated pseudotumor formation suffered from subsequent dislocations due to moderate hip abductor muscle necrosis that eventually required a second revision. In a study by Gill et al. [23] of 35 patients who received modular THAs, three patients presented with postoperative pain secondary to pseudotumor formation as a result of corrosion at the head–neck interface. Histological analysis demonstrated ALVALs of the periprosthetic tissue. Upon retrieval analysis, corrosion was seen at the trunnions in two implants. Lindgren et al. [31] described a patient with an uncemented hip prosthesis who required revision secondary to pain, swelling, and recurrent head dislocation. Intraoperatively, pseudotumor formation and local soft-tissue destruction were identified, which were suspected to result from the corrosion observed at the trunnion–taper junction. Hsu et al. [32] presented a case of pseudotumor formation secondary to corrosion at the head–neck junction in a ceramic-on-polyethylene THA. The mass also extended around the greater trochanteric bursa and enveloped the short external rotators, resulting in moderate damage to the muscle attachments. Frozen sections showed signs of chronic inflammation and synovial necrosis. Stahnke and Sharpe [33] described a patient who presented with a large (12-cm diameter) pseudotumor and abductor muscle damage. Intraoperatively, metal wear was identified at the trunnion and histological analysis showed the presence of ALVALs.

**Table 3:** Summary of studies reporting local tissue reactions

References	Number of hip implants	Implant description	Results/findings
Cooper et al. [30]	10	MoP THA	Black, flaky material at taper base, fluid collection, hypertrophic synovial tissue, and pseudotumors
Gill et al. [23]	35	MoP THA	Psuedotumor in three patients, with aseptic lymphocyte-dominated vasculitis-associated lesions in peri-prosthetic tissue
Lindgren et al. [31]	1	Uncemented MoP THA	Local soft-tissue destruction, pseudotumor
Hsu et al. [32]	1	Ceramic on polyethylene THA	Psuedotumor formation, damage to short external rotators. Chronic inflammation and synovial necrosis seen on frozen sections
Stahnke and Sharpe [33]	1	MoP THA	Pseudotumor formation (12 cm diameter) and aseptic lymphocyte-dominated vasculitis associated lesions. Patient had accompanying loss of abductors and a pelvic discontinuity

*MoM* metal-on-metal, *MoP* metal-on-polyethylene

### Gross trunnion failure

The mechanisms leading to gross trunnion failure are largely unknown; however, its manifestations have been reported in several studies (Table 4).

**Table 4:** Summary of studies reporting gross trunnion failure

References	Number of hip implants	Implant description	Results/findings
Banerjee et al. [2]	5	Five different stem designs	Trunnions exhibited either a fracture or gross loss of material volume upon revision
Hohman et al. [34]	1	First-generation proximally coated titanium cementless stem	Damaged liner insert, an intact femoral head, diffuse metallic debris, and excessive wear of the trunnion
Pansard et al. [35]	1	12-mm/14-mm trunnion	Abnormal mobility between the femoral head and the trunnion, with severe damage at the taper. Macroscopic pitting and wear
Botti et al. [36]	1	Modular locking femoral prosthesis	Trunnion fracture. Pitting and crevice corrosion on fracture surface. Black metallic debris on proximal femoral stem

Banerjee et al. [2] reported a case series of five patients who presented with gross trunnion failure after primary THA, necessitating revision. In this report, gross trunnion failures were defined as trunnions that exhibited a fracture or gross loss of material volume upon revision. All patients received modular components from five different manufacturers with femoral head diameters ranging from 28- to 40-mm. The authors were unable to locate a common link among these patients. However, it was suspected that the trunnion failures were due to a combination of patient, surgical, and component factors such as comorbidities, trunnion composition, skirted necks, formation of heterotopic ossification, and component damage during insertion. Hohman et al. [34] described a case of a THA that failed 3 years postoperatively. Intraoperative findings included a damaged liner insert, an intact femoral head, diffuse metallic debris, and excessive wear of the trunnion. Additionally, Pansard et al. [35] reported a patient who had unexplained pain at 2 years following THA and was found to have asymmetric fit of the trunnion and head on radiographic imaging. During revision, abnormal mobility was found between the femoral head and the 12-mm/14-mm trunnion, with severe damage at the taper. Further examination of the trunnion showed macroscopic pitting and wear. Histological analysis of periprosthetic tissues showed inflammatory infiltration of lymphocytes, plasmocytes, and multinucleate giant cells containing fine black particles indicative of metallosis. Botti et al. [36] presented a patient who suffered from trunnion fracture 14 years after the index THA, with no preceeding trauma. Upon retrieval analysis, it was determined that the fracture surface exhibited characteristics of pitting and crevice corrosion. Black metallic debris was also observed on the proximal region of the femoral stem, which was suspected to be caused by repetitive contact of the fractured surfaces.

## Conclusion

Trunnionosis is a well-known cause of failed total hip arthroplasties. While contributing elements may include corrosion, mechanical stress, and particular implant designs, the exact origin of trunnionosis remains poorly understood. There is growing evidence to suggest that orthopedists should be aware of soft-tissue reactions as well as signs of trunnion failure in patients who have received a THA. Moreover, continued analysis of periprosthetic tissues and retrieved implants is fundamental for understanding how patient factors influence the cellular response to corrosion debris.
